# The influence of sex hormones on renal cell carcinoma

**DOI:** 10.1177/17588359241269664

**Published:** 2024-08-21

**Authors:** Michael Ladurner, Andrea Katharina Lindner, Peter Rehder, Gennadi Tulchiner

**Affiliations:** Department of Urology, Medical University of Innsbruck, Innsbruck, Austria; Department of Urology, General Hospital Hall in Tirol, Hall in Tirol, Austria; Department of Urology, Medical University of Innsbruck, Innsbruck, Austria; Department of Urology, Medical University of Innsbruck, Anichstrasse 35, Innsbruck 6020, Austria

**Keywords:** androgen, hormone signalling axis, immune checkpoint inhibitors, oestrogen, renal cell carcinoma, RNA, sex, sex hormones, tumour microenvironment

## Abstract

Kidney cancer is a common malignancy that constitutes around 5% of all cancer cases. Males are twice as likely to acquire renal cell carcinoma (RCC) compared to females and experience a higher rate of mortality. These disparities indicate that sex hormone (SH)-dependent pathways may have an impact on the aetiology and pathophysiology of RCC. Examination of SH involvement in conventional signalling pathways, as well as genetics and genomics, especially the involvement of ribonucleic acid, reveal further insights into sex-related differences. An understanding of SHs and their influence on kidney cancer is essential to offer patients individualized medicine that would better meet their needs in terms of prevention, diagnosis and treatment. This review presents the understanding of sex-related differences in the clinical manifestation of kidney cancer patients and the underlying biological processes.

## Introduction

Renal cell carcinoma (RCC) represents around 5% of newly diagnosed cancer cases in men and 3% in women, ranking the sixth most common malignancy in males and tenth in females with a more than doubled incidence over the past half-century in the developed world. Three main histological subtypes can be distinguished, with the most common being clear cell RCC (ccRCC), followed by papillary RCC (pRCC) and chromophobe RCC (chRCC).^1^ Most cases are discovered incidentally during imaging, leading to a survival rate being dependent on the stage at diagnosis. General risk and modifiable risk factors include obesity, smoking, poorly controlled hypertension and renal failure.

Underlying genetic predispositions significantly contribute to tumour development, further course of the disease and even therapy response. An example that has fundamentally changed the therapy approach in RCC is the finding of mutations in the gene. The VHL gene mutations are associated with the Hypoxia-inducible factor (HIF)/vascular endothelial growth factor (VEGF) signalling pathway, which results in the overexpression of VEGF and platelet-derived growth factor (PDGF) receptors.^2,3^ This finding leads to the notably angiogenic characteristic of RCC and to the development of targeted therapies for metastasized disease, such as tyrosine kinase inhibitors (TKIs), mammalian target of rapamycin (mTOR) inhibitors and recombinant humanized monoclonal IgG1 antibodies that oppose VEGF.^4–11^

The highly immunogenic nature is a further characteristic of RCC,^12^ being the basis for already outdated therapeutic approaches with interleukin (IL)-2 and interferon-α (IFN-α) as well as the to date cutting-edge RCC treatment with immune checkpoint inhibitors (ICIs).^10,13–20^

In addition to characteristics such as vascularization and immunogenicity, emerging evidence highlights the importance of sex hormone (SH) signalling in various solid tumours. Previous pan-cancer analyses have identified sex-specific characteristics in the tumour microenvironment (TME), impacting tumour mutation burden (TMB), immune cell counts, immune checkpoint genes and related functional pathways in the TME.^21^ SH and their corresponding receptors, including the androgen receptor (AR), oestrogen receptor (ER) and progesterone receptor (PR), act as ligand-dependent transcription factors and play critical roles in cellular growth and differentiation, both in neoplastic and non-neoplastic states.^22^ Moreover, there is increasing evidence that these receptors can also stimulate gene expression through pathways that are independent of ligand interactions.^23^ This role is well documented in hormone-dependent organs such as breast, prostate and gynaecologic cancers^24–26^ and the significance of these pathways is evident in the efficacy of hormone withdrawal treatments using specific inhibitors.^26–28^ Furthermore, exposure to diethylstilbestrol (DES), a synthetic oestrogen or environmental chemicals mimicking androgens, increases the risk of clear cell adenocarcinoma of the cervix or vagina in women and promotes the proliferation of prostate cancer in men, respectively.^29–31^

However, there is increasing attention to intriguing sex-based disparities in cancer incidence, particularly the higher rates observed in men compared to women, affecting nonreproductive organs.^32–36^ AR activation mediated by testosterone and its derivatives has been described as a stimulator of tumour cell proliferation and migration, which may contribute to the higher incidence of many cancers in men.^37–42^ Conversely, female SHs oestrogen (E2) and PR may inhibit tumour cell proliferation and migration, primarily by inducing apoptosis, thereby reducing the risk of tumour development in certain contexts. Notably, the effects of E2 may vary or even oppose each other depending on the forms of ER (ER-α vs ER-β), its genetic variations (ERα36) and, finally, the target organ.^43–47^

As with many neoplasms, RCC is more common in men, suggesting that underlying sex-specific pathophysiology and pathways may underlie the epidemiological differences in tumour development.^48,49^ Initial findings indicate that hormones also play a role in the development and progression of RCC.^50^ The implication of the role of steroid receptor signalling pathways has been described similarly, as regression of metastatic RCC during the administration of progestin or androgen was reported.^51^ Other sex-related hormonal factors, like age at first childbirth, parity, oral contraceptive use and the condition after hysterectomy, have even more interestingly shown to affect the risk of RCC.^52–54^ However, these epidemiological studies present a challenge in differentiating between sex, which pertains to biological and physiological attributes, and gender, which refers to socially constructed attributes.^55–57^

Nevertheless, it has shown to be crucial to elucidate and gain a more detailed understanding of the hormonal-molecular pathway mechanisms involved in RCC development and progression to further promote individualized tumour prevention and treatment strategies. Furthermore, although the role of ICI in the primary therapy setting will not as quickly be displaced, potential as well as supportive or alternative treatments will be discussed. As sex therefore entails both individual genetic and pathophysiological attributes, we aim to describe, understand and analyse the role of sex itself and SH in kidney cancer formation.

## Methods

Literature research was conducted between January 2023 and September 2023 to identify studies reporting on the association between SH and RCC development. The search was performed using commonly used databases, including PubMed, Medline and Google Scholar. The study language was limited to English. The following medical subject heading terms were used to identify relevant results: ‘kidney cancer’, ‘renal cell cancer’, ‘metastatic RCC’, ‘tumour microenvironment’, ‘sex hormones’, ‘steroid receptors’, ‘oestrogen’, ‘testosterone’, ‘progesterone’, ‘androgen’, ‘oestrogen receptor’, ‘androgen receptor’, ‘progesterone receptors’, ‘RNA’, ‘tyrosine kinase inhibitor’, ‘immunotherapy’ and ‘checkpoint inhibitor’. Thereafter, 38 on general topics and 151 studies that investigate specific aspects of the research area were included in this review.

## SHs and signal transduction pathways in renal cell cancer

Signal transduction pathway-mediated processes refer to the series of molecular events that transmit signals from the cell surface to the nucleus, leading to specific cellular responses. These processes play a fundamental role in various biological functions, including cell growth, differentiation, proliferation and survival. Interaction with this mechanism by different signal molecules like AR, ER, PR and their ligands causes activation or inactivation of downstream signalling components.^58,59^ Here, we present evidence strongly suggesting that the interplay between the SH axis and signalling pathways implicated in oncogenesis, observed in other cancer types, may also have relevance in the development of RCC (Table 1).

**Table 1.** Studies characterizing sex hormone interactions with signalling pathways during cross-talk in renal cell cancer.

Sex hormone axis	Receptor	Regulation	Pathway	Effect	Reference
Androgen	AR	↑	HIF2α/VEGF/VHL	• Tumour progression • Cell migration and invasion	(60)
	AR	↑	PI3K/AKT/NF-κB/CXCL5	• Tumour progression • Vascular endothelial cell proliferation and recruitment	(61)
	AR	↑	Neutrophil/c-Myc	• Tumour cell proliferation	(62)
	AR	↑	STAT5 phosphorylation	• Tumour cell proliferation	(63)
Oestrogen	ER-α	↑	VHL/HIF-1α/p53	• Tumour cell proliferation	(64)
	ER-α	↑	VEGFa/HIF-2α	• Tumour cell proliferation	(65)
	GPER	↑	PI3K/AKT/MMP-9	• Tumour progression • Cell migration	(66)
	ER-β	↓	AKT/ERK/JAK/Bid → Caspase-3, -8, -9	• Enhancement of tumour apoptosis	(67)
	ER-β	↑	TGF-β1/SMAD3	• Tumour progression • Cell migration	(68)
	ER-β	↑	Angiopoietin-2/Tie-2	• Vascular endothelial cell proliferation and recruitment	(69)

AR, androgen receptor; Bid, BH3 interacting-domain death agonist; ER, oestrogen receptor; ERK, extracellular signal-regulated kinase; GPER, G-protein coupled oestrogen receptor; HIF-2α, hypoxia-inducible factor-2α; JAK, janus kinase; MMP-9, matrix metalloproteinase-2; NF-κB, nuclear factor-κB; PI3K, phosphoinositide 3-kinases; pVHL, von Hippel-Lindau protein; RCC, renal cell carcinoma; TGF-β1, transforming growth factor beta 1; VEGF, vascular endothelial growth factor; ↑, promoting effect; ↓, inhibiting effect.

### Androgens

The androgen signalling axis is described as a key player in the development and progression of prostate cancer,^26^ which is recognizably already influenced by sex in its primary development. Yet, an increasing number of studies suggest a potential contribution in RCC as well, which is gender-independent at first glance. For instance, a varying AR expression (15%–55%) in RCC with mixed correlations to outcomes was revealed.^70–74^ A noteworthy correlation between AR expression and lower pathological stage and grading at diagnosis, as well as subsequent better outcomes, has been described.^70,71,75,76^ Controversially, studies have indicated worse oncological prognosis and overall outcome in correlation to AR expression^72,73,77^ as well.

In this context, the AR has been identified as a potential co-regulator of the HIF2a/VEGF signalling pathway.^78^ Its activation induces HIF2α/VEGF expression in RCC tumour tissue, subsequently promoting RCC progression. In a preclinical mouse model, targeting AR with AR degradation enhancers showed an effectively suppressed RCC progression, indicating a promising therapeutic approach.^60^ Guan et al. described a further pro-angiogenic effect of AR by demonstrating its function as a regulator of the PI3K/AKT → NF-κB → CXCL5 signalling pathway, which is known to influence RCC progression and endothelial cell recruitment. The group highlighted the involvement of NF-κB, a chemokine responsible for tumourigenesis and inflammation in cancer cells. NF-κB has been suggested to be an additional potential mediator in the PI3K/AKT signalling pathway, contributing to RCC progression.^61^

RCC is widely recognized as a typical ‘hot tumour’, characterized by an abundant infiltration of CD8+ T cells in the TME.^79–81^ While a higher infiltration of CD8+ T cells predicts a better prognosis in many cancers due to their cytotoxic function,^82–84^ this correlation does not apply to RCC patients.^85^ However, CD8+ T cells have distinct subtypes, with conventional cytotoxic CD8+ T cells having an anticancer role, whereas exhausted CD8+ T cells become dysfunctional. Studies investigating sex bias have identified the exhausted and dysfunctional state of CD8+ T-cells in various cancers, including bladder, prostate and liver cancers. In these contexts, the AR has been implicated both as a transcription factor promoting exhausted CD8+ T-cell formation and as an inhibitor of CD8+ T-cell function, activity and stemness.^86–88^ Investigations into the differences in the TME of RCC between male and female patients have revealed that male RCC TME exhibits higher infiltration and exhaustion of CD8+ T cells compared to females. Additionally, the crucial role of the androgen-AR axis in inducing CD8+ T-cell exhaustion in RCC could be shown.^89^ The presence of intratumoural neutrophils in RCC has been associated with a negative prognosis in RCC as well. High-grade RCC patients exhibited a higher degree of neutrophil infiltration in tumour tissue compared to low grade, indicating the potential role of cancer-induced immunosuppression in promoting cancer progression through the modulation of neutrophils.^90^ Focusing on N2 neutrophils, which are known to be involved in carcinogenesis, angiogenesis and immunosuppression, Song et al.^62^ described their ability to promote RCC proliferation by upregulating AR expression via the AR-c-Myc signalling pathway.

Moreover, high levels of dihydrotestosterone (DHT) receptors were found more often in higher staged RCC tumours.^91^ In this context, He et al. conducted an experimental model demonstrating that transfected normal human kidney cells with AR with a subsequent exposure to a carcinogen, resulted in a higher incidence of larger cell colonies and growth. Furthermore, inoculating functional AR into stable RCC cell lines resulted in increased tumour proliferation.^60^ Pak et al. observed that treatment with DHT led to an increase in cell proliferation in both AR-positive and AR-negative RCC cells, depending on the concentration of DHT. The study also showed a dose-dependent rise of STAT5 in RCC cells following DHT treatment. Yet, as AR was knocked down with small interfering ribonucleic acid (siRNA), there was a reduction in cell proliferation specifically in AR-positive cells. Interestingly, the decrease in phosphorylated STAT5 levels after AR knockdown mirrored these findings, implicating STAT5 activation as a potential mechanism behind DHT-induced RCC cell growth.^63^ These observations provide evidence that the androgen axis affects cell proliferation and migration and therefore must be relevant in the context of RCC development.^92–95^

### Oestrogens

The E2 signalling axis has been shown to influence the development of RCC significantly as well. Two distinct forms of ER, ER-α and ER-β, are present in normal renal tissue.^96^ ERs have furthermore already been detected in stromal tumours, cystic nephromas and angiomyolipoma.^97–102^ ERs in RCC seem to be highly variable in their expression with controversial data. Earlier studies indicate a high ER presence (30%)^100^ followed by more recent observations, describing a low ER distribution (1.1%). E2 is suggested to affect RCC cell viability, DNA damage, oxidative stress, nuclear factor phosphorylation, clearly influencing tumour growth and additionally may affect the balance between autophagy and apoptosis in RCC.^67,103^

#### Oestrogen receptor-α

Genetic variations within the ER-α gene have a notable role in RCC development. An exploratory analysis of 113 RCC cases revealed differences in genotype distribution at codon 10 on exon-1 of the ER-α gene compared to healthy individuals.^104,105^ ERα36 is known to be another splice variant, found in the cytoplasm and plasma membrane. Elevated ERα36 levels in these locations are linked to adverse prognostic factors in RCC, including poor disease-free survival, larger tumour size and advanced clinical stages.^46,106^

A complex underlying interaction in the RCC formation involves the interplay between ER-α, VHL, HIF-1α and p53. ER-α is assumed to be a target for proteasomal degradation by the tumour suppressor von Hippel-Lindau protein (pVHL) E3 ligase. Overexpression of pVHL suppresses ER-α in RCC, while pVHL downregulation increases ER-α expression.^64^ Elevated ER-α expression has been found to enhance the activity of the HIF-1α transcription factor. In VHL-deficient cells, the expression of both ER-α and HIF-1α persists, and blocking ER-α using its inhibitor can effectively inhibit the proliferation of VHL-deficient cells. Notably, the anti-proliferative effect of faslodex, an ER-α inhibitor, in VHL-deficient cells by inducing the expression of p53 could be demonstrated.^64^ ER-α furthermore promotes the transcription of growth-related factors, driving gene expression, mitosis, proliferation, cancer development and tumour progression.^54^

The G-protein-coupled oestrogen receptor (GPER), distinct from nuclear ER, also plays a role in oestrogen-dependent development and progression of cancers, including RCC.^107–110^ RCC cell lines express GPER abundantly and its activation promotes RCC cell migration and invasion by upregulating matrix metalloproteinase-2 (MMP-2) and MMP-9, as well as activating downstream signalling pathways, particularly MAPK and PI3K/AKT, promoting cell migration via the PI3K/AKT/MMP-9 pathway.^66^

#### Oestrogen receptor-β

The role of ERβ remains controversial. While some studies show an inhibitory effect on cell proliferation, others suggest that ERβ expression may promote cancer development.^111–113^ However, initial investigations suggest that ER-β signalling appears to have a tumour-suppressive role in RCC^114^ characterized by anti-proliferative functions, inhibition of migration, suppression of invasion and enhancement of apoptosis.^54,67,103^

The inhibitory effects of oestrogen via ER-β activation in RCC involve dampening downstream hormone signalling, including AKT, extracellular signal-regulated kinase (ERK) and janus kinase (JAK) activation, while increasing the expression of apoptotic genes such as BH3 interacting-domain death agonist (Bid), Caspase-3, Caspase-8 and Caspase-9.^54,67^ Conversely, subsequent investigations revealed the contrary role of ER-β in RCC. Clinical data showed increased ER-β expression in advanced-stage or high-grade tumours, correlating with unfavourable survival outcomes and reduced disease-free survival for RCC patients.^68,69,115,116^ ER-β has been identified as a promoter of RCC cell invasion through the augmentation of the TGF-β1/SMAD3 signalling axis. Targeting this pathway with anti-oestrogens or TGF-β receptor inhibitors effectively reduced RCC tumour growth and invasion.^68^ ER-β was also found to regulate Angiopoietin-2 (ANGPT-2)in RCC cells through oestrogen response elements (EREs) on the ANGPT-2 promoter. The escalated ANGPT-2 levels in RCC cells exert a stimulatory influence on angiogenesis by engaging and phosphorylating the Tie-2 receptor, leading to the formation of HUVEC tubes. Targeting the ER-β/ANGPT-2/Tie-2 pathway with faslodex-enhanced RCC sensitivity to the TKI sunitinib treatment emerges as a promising therapy strategy.^69^ ER-β signalling has been implicated in inducing the VEGFa/HIF2α pathway as well.^65^ Infiltrating immune cells, particularly T cells, can modify ER-β expression and can promote RCC invasion. T-cell co-cultures with RCC cells resulted in elevated levels of T-cell-attracting factors, including IFN-γ, C-C motif chemokine ligand (CCL) 3 and CCL5, suggesting the establishment of a positive regulatory feedback mechanism. Simultaneously, infiltrating T cells appears to contribute to RCC cell invasion by influencing ER-β expression and concurrently suppressing the expression of DAB2IP. Intriguingly, the suppression of DAB2IP could subsequently reverse the T-cell-mediated promotion of RCC cell invasion.^117^ The interaction in this course concerning immunotherapy treatment with the checkpoint inhibitor nivolumab in RCC patients has been recently described. This therapeutic approach, which elicits immunomodulatory effects on neutrophils, has been found to induce alterations in the expression of sex SH, particularly oestrogen, during the treatment in patients with mRCC.^118^ Notably, an intriguing observation emerged with the administration of TKIs sunitinib and axitinib, leading to increased expression and stability of ER-β in RCC cell lines.^119,120^

### Progesterone

Progesterone signalling axis has also been shown to potentially influence the development of RCC. PR expression is observed in normal and carcinomatous kidney tissue, with varying levels in different histological RCC subtypes.^51,121,122^ PR presence is reported in benign renal tumours and metaplastic nodules,^121^ at a high rate than in malignant tumours of the kidney. Higher levels of expression were notably observed in cases of renal oncocytoma (RO).^60^ Literature also suggests that PR demonstrates nuclear reactivity in a range of 10%–50% of tumour cells across all RO cases. Interestingly, up to 20% higher PR expression has been described in chRCC. In comparison, non-neoplastic renal tissue displays scattered stromal cells and tubular cells that show reactivity for both ER and PR, as in comparison fewer than 1% of RCC cases show reactivity for these receptors.^123^

Progestin and adipoQ receptor 5 (PAQR5), a membrane-bound PR, can activate downstream signalling pathways affecting various cellular processes, including cell proliferation, migration and invasion. In RCC, decreased expression of PAQR5 is linked to tumour stage, cancer grade, lymph node invasion and distal metastasis, all in all suggesting a role in RCC progression. While the precise mechanisms underlying PAQR5s regulation of downstream signalling pathways remain unclear, its functions are thought to align with pathways related to ribosome function, focal adhesion and intracellular signal transduction pathways such as PI3K-AKT, MAPK and mTOR.^124^ Progesterone Receptor Membrane Component 1 (PGRMC1) is another member of the membrane-associated PR protein family, which has been shown to distribute key functions in various cancer types, including RCC.^125^ Elevated PGRMC1 levels in RCC tissues are correlated with higher tumour stage and are more common in poorly differentiated tumours. High expression of PGRMC1 in RCC tissue compared to adjacent non-cancerous tissues evinces its potential utility as a diagnostic and prognostic biomarker for RCC, as it has already demonstrated the ability to influence cancer cell susceptibility to chemotherapy, further emphasizing its correlation with tumour malignancy and progression.^125^

## SHs and RNA-mediated processes in RCC

Noncoding RNA (ncRNA) and their influence on regulatory processes open up a further field of interest concerning hormone-dependent modulation of RCC initiation and progression at a molecular level. NcRNA encompasses approximately 90% of the human transcriptome and does not encode proteins. Its involvement in tumour initiation and progression has gained significant attention in recent years.

**Figure 1.**
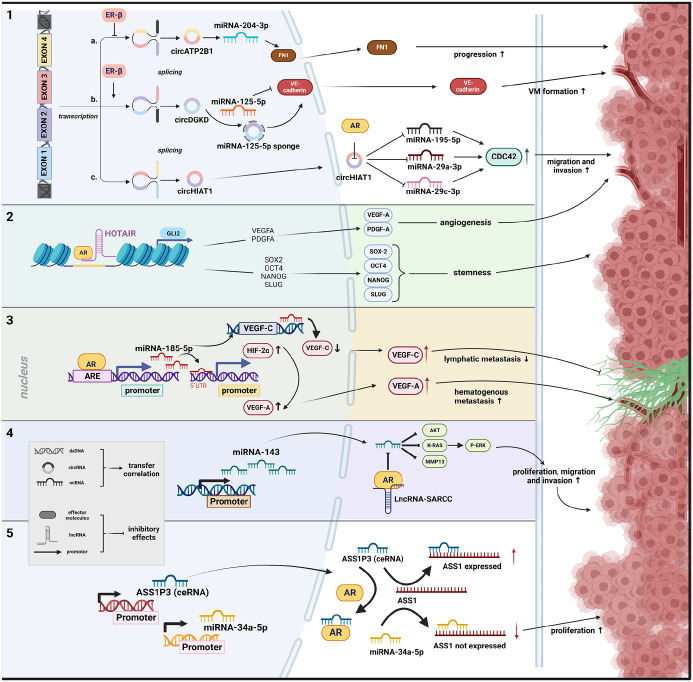
Schematic representation of potential interaction mechanisms between sex hormones and ncRNA regarding RCC formation. (1) Function via altering circRNA with inhibiting effect of ER-β on transcription of circRNA,^116^ promoting effect of ER-β on transcription of circRNA^120^ and direct effect of AR on circRNA function.^137^ (2) The interaction between AR and lncRNA with chromatin has a synergistic effect on the activities that affect the intrachromosomal genes located nearby.^133^ (3) The effect of AR on miRNA expression occurs through the direct binding of its candidate AREs in the promoter region.^136^ (4) The function and stability of a protein is influenced by its interaction with lncRNA, thereby impacting interactions between the protein and miRNA.^134^ (5) Direct interaction with AR can regulate the ceRNA activity, resulting in an increased effect of the competing miRNA.^132^ Source: Created with BioRender.com. ARE, androgen response elements; ceRNA, competitive endogenous RNA; circRNA, circular RNAs; lncRNA, long noncoding RNA; miRNA, microRNA.

MicroRNA (miRNA) consists of a small RNA molecule that is capable of binding to the 3′-untranslated region (3′-UTR) of target gene transcripts, leading to translational repression or mRNA destabilization. Long noncoding RNA (lncRNA) exceeding 200 nucleotides in length has also been shown to play a regulatory role in cancer biology.^126^ These types of RNA can either act as oncogenes or tumour suppressor genes, exerting influence by modulating diverse signalling pathways.^127^ Another category of ncRNA, referred to as pseudogenes which are non-functional duplicates of genes, has been identified as a significant contributor to cancer^128^ and may act as competitive endogenous RNAs (ceRNA).^129^ Finally, circular RNAs (circRNA) constitute a newly discovered group of ncRNAs, primarily formed as loop structures at the exons due to non-canonical splicing. Recent data suggest that aberrant circRNA expression is linked to the onset and progression of diseases, particularly various types of human malignancies.^130,131^ The following chapters will present current findings on the connection between ncRNA and the signalling pathways of SH, analysing their potential involvement in the progression of RCC (Table 2 and Figure 1).

**Table 2.** Studies characterizing sex hormone interactions with ncRNA during cross-talk in renal cell cancer.

Sex hormone axis	HR/ncRNA interaction	ncRNA effect	Pathway	Regulation	Effect on RCC	Reference
Androgen	AR/ASS1P3	↓ ASS1P3	miRNA-34a-5p/ASS1	↑	• Tumour proliferation • Tumour progression	(132)
	AR/HOTAIR	↑ HOTAIR	GLI2/VEGFA + PDGFA/CSC (SOX2, OCT4, NANOG, SLUG)	↑	• Tumour angiogenesis • Cancer stemness	(133)
	lncRNA-SARCC/AR/miRNA-143-3p	AR ↓ miRNA-143-3p lncRNA-SARCC ↓ AR	AKT; MMP-13; K-RAS; P-ERK	↑	• Tumour cell migration • Tumour proliferation • Tumour progression	(134)
	lncRNA-SARCC/AR	lncRNA-SARCC modulates the AR under different oxygen conditions	HIF-2α/C-MYC	↑↓	• Suppresses tumour progression under hypoxia • Induces tumour progression under normoxia	(135)
	AR/miRNA-185-5p	AR ↑ miRNA-185-5p expression miRNA-185-5p ↓ VEGF-c and ↑ HIF-2α/VEGFA expression	VEGF-c HIF-2α/VEGFA	↑↓	• Increases haematogenous metastasis • Decreases lymphatic metastasis	(136)
	AR/circHIAT1	AR ↓ circHIAT1 resulting in deregulating miR-195-5p/29a-3p/29c-3p expressions, which ↑ CDC42 expression	miRNA-195-5p, miRNA-29-3p, miRNA-29-3p/CDC42	↑	• Tumour cell migration • Tumour cell invasion	(137)
	AR /miRNA-145	AR ↓ miRNA-145	HIF-2α/VEGFA, MMP-9, CCND1	↑	• Tumour cell invasion • Tumour cell proliferation	(138)
Oestrogen	ER-β/circATP2B1	ER-β ↓ the expression of circATP2B1 CircATP2B1 ↓ miRNA-204-3p, which caused the ↑ expression of FN1	miRNA-204-3p/FN1	↑	• Tumour cell migration • Tumour progression	(116)
	ER-β/HOTAIR	ER-β ↑ HOTAIR expression HOTAIR ↓ various miRNA and their suppressive effect on different oncogenes	miRNA-138/ADAM9 miRNA-204/CCND2 miRNA-217/VEGFA, VIM, ZEB1, ZEB2 miRNA-200c/ZEB1, ZEB2	↑	• Tumour cell migration • Tumour proliferation • Tumour cell invasion	(115)
	lncRNA-ECVSR/ER-β	lncRNA-ECVSR ↑ ER-β miRNA stability ER-β → Hif2-α ↑ Hif2-α → CSC phenotype and VM formation ↑	HIF-2α/VM formation	↑	• Tumour angiogenesis	(119)
	ER-β/circDGKD	ER-β → circDGKD expression ↑ CircDGKD sponge miRNA-125-5p miRNA-125-5p → VE-cadherin ↑	miRNA-125-5p/VE-cadherin	↑	• Tumour angiogenesis	(120)

AR, androgen receptor; ASS1, argininosuccinate synthase 1; CDC42, cell division cycle 42 protein; circATP2B1, circular RNA ATPase plasma membrane transporter 2B1; circDGKD, circular RNA DGKD; CSC, cancer stem cell; ER, oestrogen receptor; FN1, fibronectin 1; HOTAIR, HOX transcript antisense intergenic RNA; K-RAS, Kirsten rat sarcoma viral oncogene; lncRNA, long noncoding RNA; miRNA, micro RNA; ncRNA, noncoding RNA; PDGFA, Platelet derived growth factor subunit A; RCC, renal cell carcinoma; VE-cadherin, vascular endothelial cadherin; VEGF, vascular endothelial growth factor; VM, vasculogenic mimicry; ↑, promoting effect; ↓, inhibiting effect.

### Androgens

Argininosuccinate synthase 1 (ASS1) expression showed a negative correlation to tumour staging, indicating a reduced ASS1 expression during tumour progression. Recent studies have additionally highlighted the significance of decreased ASS1 activity in promoting tumour growth. Immunohistochemical staining of 40 each primary RCC and adjacent normal renal tissue samples demonstrated a lower ASS1 expression in RCC tissue. Following this, one could implicate that decreased ASS1 expression is associated with a poorer prognosis in RCC, highlighting the possible role of ASS1. Increasing AR expression in vitro led to decreased ASS1 expression, promoting cell proliferation. Conversely, AR knockdown resulted in increased ASS1 expression. In this context, miRNA-34a-5p has been identified as a regulator of ASS1, capable of negatively modulating its expression. Notably, ASS1P3 has been indicated as a pseudogene, which could function as a ceRNA, to modulate the expression of its corresponding gene, ASS1, by competing with miRNA-34a-5p. Reduced ASS1P3 expression inhibited ASS1 by miRNA-34a-5p, resulting in increased cell proliferation. Although a direct correlation between AR and ASS1P3 expression was not detected by the authors, it was postulated that AR may physically interact with ASS1P3, thereby impeding the interaction between ASS1P3 and miRNA-34a-5p.^132^ Bai et al.^133^ investigated the relationship between the lncRNA known as HOX transcript antisense intergenic RNA (HOTAIR) and AR in human ccRCC. HOTAIR is a trans-acting lncRNA located on chromosome 12q13.13, with a regulatory boundary in the HOXC cluster.^139^ HOTAIR, along with other lncRNAs associated with the HOX locus, such as HOTAIRM1 and HOTTIP, plays significant roles in the development of ccRCC,^140–142^ through various mechanisms.^115,143–145^ The Hedgehog-GLI (HH-GLI) signalling pathway has been implicated in promoting cellular proliferation, differentiation, vascularization and stem cell maintenance. In RCC, GLI1/2 is activated by PI3K/AKT signalling.^146^ Here, it was shown that GLI2 serves as a target gene for both HOTAIR and AR synergistically. HOTAIR and AR cooperatively bind to the GLI2 promoter, leading to an increase in its transcriptional activity. Consequently, GLI2 and its downstream genes, including cancer stem cell (CSC) transcription factors, vascular endothelial growth factor A (VEGFA) and PDGFA, were upregulated. This upregulation promotes tumour angiogenesis and enhances cancer stemness in RCC cells both in vitro and in vivo.^133^

MiRNA-143-3p, identified as a tumour suppressor, is frequently down-regulated in various cancers, including RCC.^147–150^ Its decreased expression has been associated with the promotion of RCC cell invasion, migration and proliferation through downstream signalling molecules, including AKT, MMP-13, K-RAS and P-ERK. In a study by Zhai et al. AR influence of miRNA-143-3p expression by direct binding to its potential androgen response elements (AREs) in its promoter, thereby transcriptionally suppressing miRNA-143-3p was discovered. Additionally, a long noncoding RNA called suppressing AR in RCC (lncRNA-SARCC) was identified to directly bind and suppress the AR function by post-transcriptionally modulating the AR protein, consequently increasing miRNA-143-3p expression and suppressing the RCC progression. Consequently, expression of lncRNA-SARCC was found to be reduced in ccRCC and metastatic ccRCC compared to surrounding non-tumour and non-metastatic tissues, and this reduction correlated with a poorer prognosis in ccRCC patients. Interestingly, the authors observed that Sunitinib induces the expression of lncRNA-SARCC, thereby reducing the resistance of RCC cells to this drug.^134^ An additional suppressive effect of LncRNA-SARCC on RCC development could be revealed through its regulation of the AR/HIF-2α/C-MYC axis signalling pathway. Interestingly, the expression of lncRNA-SARCC was found to be mediated differently in response to hypoxia. Under hypoxic conditions, lncRNA-SARCC suppressed AR expression, leading to a decrease in the HIF-2α/C-MYC axis and its cell proliferating and tumourigenic effect. In return, lncRNA-SARCC expression can be transcriptionally regulated by HIF-2α through its binding to hypoxia-responsive elements on the lncRNA-SARCC promoter, suggesting the presence of a negative feedback loop.^135^ These findings provide valuable insights into the role of lncRNA-SARCC as a suppressor of RCC progression and highlight new therapeutic strategies for the treatment of RCC, specifically focusing on the regulation of AR and miRNA interactions.

Huang et al. investigated a novel interaction mechanism of AR with HIF-2α through miRNA regulation in ccRCC. Elevated AR expression was associated with increased haematogenous metastasis to the lung but reduced lymphatic metastases, through enhanced miRNA-185-5p expression by binding to its promoter region, leading to the suppression of vascular endothelial growth factor C (VEGF-C). Conversely, AR-mediated upregulation of miRNA-185-5p promoted HIF-2α/VEGF-A expression. This unique interplay between AR, miRNA-185-5p, VEGF-C and HIF-2α/VEGF-A highlighted AR’s dual role in facilitating or inhibiting ccRCC metastasis.^136^ In addition, AR has been found to exert differential regulation on VEGF-A and VEGF-C in VHL wild-type ccRCC, depending on the oxygen conditions (normoxia vs hypoxia), thereby impacting the metastasis processes in distinct ways. Under normoxic conditions, the down-regulation of miRNA-185-5p results in the up-regulation of both VEGF-A and VEGF-C. Conversely, in a hypoxic environment, the upregulation of miRNA-185-5p leads to a decrease in both VEGF-A and VEGF-C expression. However, the activation of HIF-2α in hypoxia leads to the transcriptional upregulation of VEGF-A, outweighing miRNA-185-5p’s down-regulation, resulting in elevated VEGF-A expression.^151^

Literature also reveals another study describing showing the link between AR and miRNAs in RCC, indicating that AR affects ccRCC cell migration and invasion by changing circHIAT1/miRNA-195-5p/29a-3p/29c-3p/cell division cycle 42 protein (CDC42) signalling. Suppression of circulating RNA circHIAT1 by AR resulted in altered miRNA-195-5p/29a-3p/29c-3p expression, which increased CDC42 expression, leading to intensified cell migration and invasion.^137^

It has been shown that AR can bind to the ARE on the promoter region of miRNA-145, leading to the reduced ability of p53 to induce miRNA-145 expression. MiRNA-145 normally acts to suppress the expression of HIF-2α, VEGF, MMP9 and CCND1, which are key factors involved in RCC progression. Suppressing AR or introducing miRNA-145 mimics reduced RCC progression, regardless of VHL status. In a preclinical RCC mouse model, miRNA-145 mimic administration effectively suppressed RCC progression.^138^

### Oestrogens

As a transcription factor, ER-β can bind to specific DNA sequences in the promoter regions of target genes, either activating or repressing their transcription. ER-β was identified as a suppressor of circRNA ATPase plasma membrane transporter 2B1 (circATP2B1) expression by directly binding to the 5′ promoter region of its host gene ATPase plasma membrane Ca2+ transporting 1 (ATP2B1), which encodes circATP2B1. CircATP2B1 is implicated in regulating miRNA-204-3p in ccRCC cells, significantly increasing miRNA-204-3p by circATP2B1 addition. Moreover, circATP2B1 may function as a so-called ‘reservoir’ to stabilize miRNA-204-3p expression, as it interacts directly with miRNA-204-3p. This interplay results in elevated fibronectin 1 (FN1) expression in ccRCC cells, as miRNA-204-3p directly targets FN1 mRNA’s 3′ UTR, suppressing FN1 protein expression. Inhibition of miRNA-204-3p increases FN1 expression, while miRNA-204-3p overexpression decreases FN1 levels in ccRCC cells. Analysis of the ccRCC patient survival data from the Cancer Genome Atlas indicates worse overall survival (OS) for patients with elevated ER-β and FN1 expression, while higher miRNA-204-3p expression correlates with significantly better OS, emphasizing the clinical relevance of the ER-β/circATP2B1/miRNA-204-3p/FN1 axis in ccRCC progression.^116^ Furthermore, ER-β facilitates an increase in HOTAIR expression by binding to its promoter in RCC. Consequently, HOTAIR assumes a role in counteracting the effects of various miRNAs. Subsequently, HOTAIR counteracts the effects of several miRNAs. By antagonizing miRNA-138, (targeting ADAM9), miRNA-204 (targeting CCND2), miRNA-217 (targeting genes like VEGFA, VIM, ZEB1 and ZEB2) and miRNA-200c (targeting ZEB1 and ZEB2), it results in collective interaction with RCC cell, proliferation, migration and invasion.^115^

Higher ER-β expression correlates with elevated VE-cadherin, a pivotal adhesion molecule in vasculogenic mimicry (VM) formation. VM is a process where tumour cells mimic blood vessel-like structures to secure nutrients and oxygen, potentially contributing to ccRCC progression and metastasis.^119^ One supposed mechanism by which sunitinib promotes VM formation in RCC cells involves the modulation of lncRNA called lncRNA-ECVSR. Sunitinib treatment can increase the expression of lncRNA-ECVSR, which enhances the stability of ER-β mRNA. This increased ER-β expression can then function via transcriptional up-regulation of Hif2-α by binding to ERE, specifically to ERE1, in the promoter region of Hif2-α. Hif2-α, in turn, promotes the CSC phenotype, which is associated with increased VM formation. The sunitinib/lncRNA-ECVSR-increased ERβ expression can transcriptionally regulate lncRNA-ECVSR expression via a positive-feedback loop, further enhancing the effects of sunitinib on VM formation.^119^

TKI-induced ER-β transcriptionally up-regulates the circular RNA DGKD (circDGKD), which functions as a miRNA-125-5p sponge. MiRNA-125-5p interacts with the 3′ UTR of VE-cadherin mRNA, leading to its degradation or translational inhibition. In RCC, miRNA-125-5p down-regulation results in increased VE-cadherin expression, promoting VM formation. Targeting circDGKD tempers sunitinib-induced RCC VM formation, reduces metastasis and enhances survival in experimental animal models. The authors propose that intervening in ERβ/circDGKD signalling may enhance TKI effectiveness and offer novel combination therapies for the management of metastatic RCC.^120^

## SHs and treatment of RCC

### Androgen treatment in RCC

The potential significance of the androgen-signalling axis in the progression of RCC has led to the thought that interfering with the hormonal axis may be a potential strategy to enhance patient survival. Clinical trials are already investigating the efficacy of therapeutic agents targeting AR in RCC. The BARE trial (Blockade of Androgens in RCC using Enzalutamide, NCT02885649, www.clinicaltrials.gov, accessed on 9 September 2021) was designed to elucidate the impact of the AR inhibitor enzalutamide on tumour growth prior to surgical resection. Regrettably, the trial was prematurely terminated due to unavailability of funding.

Flutamide, a nonsteroidal anti-androgen, was also investigated in patients with RCC. Among 25 cases treated, one patient exhibited partial remission and two patients experienced a state of stabilization of disease. Nevertheless, flutamide did not demonstrate any anti-tumour activity in individuals with metastatic RCC.^122^ Enzalutamide and abiraterone acetate, a CYP17A1 inhibitor that inhibits androgen production, showed more promising results in in vivo studies, demonstrating a substantial reduction in tumour size.^152^

Knockdown of the epigenetic co-regulator lysine-specific histone demethylase 1, along with enzalutamide, slowed RCC growth and migration in a mouse model.^153^ In a patient-derived xenograft model with sunitinib-resistant RCC, AR upregulation was observed. Enzalutamide treatment led to AR degradation and decreased AR activity, resulting in effective tumour regression when combined with sunitinib.^154^ These findings highlight the importance of the androgen axis in RCC and suggest AR as a potential target for a therapeutic approach.

### Oestrogen treatment in RCC

Investigation on hormonal carcinogenesis, specifically highlighting the involvement of the SH in RCC pathogenesis, has yielded encouraging findings. Particularly outcomes in the domain of RCC therapy targeting ER remain promising, providing notable advantages for managing metastatic disease.

In a hamster model, RCC was successfully induced through the chronic administration of DES and polydiethylstilbestrol phosphate, underlining the possibly important involvement of oestrogens in RCC aetiology.^155–157^ Conversely, the inhibitory impact on tumour formation was demonstrated with the antioestrogen agent nafoxidine.^158^ In vitro investigations proposed that potentially reactive oestrogen intermediates might act as instigators of experimental nephron-carcinogenesis, inducing substantial oxidative stress within renal cells upon prolonged oestrogen exposure.^159,160^

The potential utility of tamoxifen, a selective ER modulator commonly used in breast cancer treatment, was explored as a therapeutic approach for small cohorts of patients with RCC, yielding varied outcomes. In one study, 34 patients with progressive RCC were treated with high-dose tamoxifen (100 mg/ml 2 daily) until disease progression. An overall partial response of 10%, including one complete remission, was observed. Favourable survival outcomes were noted in patients with pulmonary metastases, good performance status and prior nephrectomy.^161^

Another investigation involved 10 patients with advanced RCC treated using combined chemo-endocrine therapy comprising tegafur, a prodrug of fluorouracil, and tamoxifen. Positive responses were observed, particularly in patients with ER-positive and ER-negative tumours.^162^ A comparison study evaluated tamoxifen alone against IL-2/IFN-α therapy combined with tamoxifen. Although tamoxifen was included due to its non-toxic behaviour and potential enhancement of IL-2’s anti-tumour activity, no significant survival differences were found between treatment arms.^163^ While high-dose tamoxifen demonstrated some anti-tumour effects in specific cases, combined hormonal therapy did not confer a significant therapeutic advantage for advanced RCC. Despite this, subsequent years have seen limited research exploring the potential of hormone modulators in RCC. This is noteworthy considering the emergence of new therapeutic strategies such as TKIs and immune-based therapies, which have shown promise in metastatic RCC management.

### Progesterone treatment in RCC

Hormonal agents, like medroxyprogesterone, were found to have some effectiveness in treating metastatic RCC in early studies. However, limited data show response rates in different studies, ranging from 7% to 25%, and still need further evaluation in larger studies to confirm oncological ongoing response.^164–166^

### Sex and immunotherapy in RCC

Previous research has shown differences in immune responses, particularly anti-tumour responses, between sexes.^167,168^ Meta-analyses propose that ICIs may provide greater benefits for male cancer patients in comparison to females.^169–171^ The controversially discussed data do not necessarily apply to RCC.^172,173^ A recent comprehensive review focused on the efficacy of ICI in urological cancers, including RCC, indicating an improved OS, regardless of sex.^174^ Meanwhile, adjuvant ICI monotherapies reduce the risk of disease recurrence in women with locally advanced RCC, yet not in men. Furthermore, ranking analyses revealed distinct outcomes for RCC treatment between the sexes, suggesting that sex may influence clinical decision-making.^174^ However, there is evidence of a divergent response to ICI treatment in patients with advanced RCC, with a less marked effect observed in females compared to male patients, indicating that sex is a crucial factor in clinical decision-making.^175^

Another intriguing aspect of sex differences lies in the occurrence of adverse events associated with ICI. Immunotherapy disrupts immune balance, potentially leading to immune-related adverse events (irAEs) affecting various organ systems.^176^ Women exhibit higher innate and adaptive immune responses than men, along with an increased susceptibility to autoimmune diseases, leading to a higher risk of irAEs.^177^ Consequently, female sex has been identified as a predictive biomarker for irAE occurrence in patients treated with ICIs.^178^ Notably, a study by Unger et al.^179^ examining gender disparities in therapy responses, particularly to immunotherapy, revealed that women receiving immunotherapy exhibited a 49% greater risk of irAEs than men, with the severity of irAEs being higher among women.

Some further nuanced relationships between sex and molecular predictors of ICI response could be revealed. TMB has been associated with a positive ICI response in men with certain cancers such as melanoma, bladder, head and neck, and RCC.^180^ Conversely, other molecular markers, such as activated T-cell frequency and expression of immune checkpoint proteins, including both inhibitory (programmed cell death protein1 1 (PD-1), cytotoxic T-lymphocyte-associated Protein 4 (CTLA-4), Lag3) and stimulatory (OX40, ICOS, CD27) markers, are associated with a positive ICI response in female patients.^181^

In this context, SHs are suspected to influence the TME and underlie the differences in immune responses between men and women.^182^ E2 increases immunoglobulin production, while androgens such as dihydrotestosterone (DHT) and testosterone have been shown to reduce immune activity.^167^ Regulatory T cells (Treg) rise when E2 levels increase.^183^ Reduced levels of E2 encourage differentiation of T helper (Th) cells towards Th1, whereas higher levels of E2 promote the Th2 phenotype.^184^ Additionally, E2 is linked with heightened expression of PD-1.^183^

The persistence of androgen-induced exhaustion of CD8+ T cells may limit the efficacy of ICIs in male RCC, necessitating additional therapeutic strategies.^89^ Androgen deprivation therapy (ADT) can prevent CD8+ T-cell exhaustion in the TME and improve the efficacy of ICIs, particularly anti-PD-1 treatment.^86,87^ Combination therapy with androgen receptor inhibitors (ARi) and ICIs has shown synergistic effects in RCC in vivo, possibly because ARi reverses androgen-induced immunosuppression in the TME of male RCC.^89^ Other investigations suggest that ADT can enhance the effectiveness of immunotherapy by modulating immune cell function.^185^ Moreover, studies have shown that dendritic cells are better able to stimulate T-cell responses when ADT is administered after immunotherapy.

Nevertheless, the regulatory impact of SH on immunotherapy in cancer, particularly the association between SH and ICI in metastatic RCC, has not been thoroughly evaluated. There is a notable lack of data and the interpretation is complicated, also due to the insufficient exploration of the reciprocal effects of checkpoint inhibition on SH and vice versa. However, anti-PD-L1 therapy has been shown to significantly downregulate SH levels in male mice, but not in female mice, thereby enhancing the anti-tumour efficacy of anti-PD-L1.^186^ In contrast, an increase in estradiol and luteinising hormone (LH)/follicle-stimulating hormone (FSH) ratio in male patients receiving nivolumab monotherapy for metastatic RCC has been recently reported and an association between progression free survival (PFS) and objective response rate (ORR) with increased LH/FSH ratio during nivolumab therapy has been demonstrated.^118^

Additionally, survival outcomes among RCC patients may be influenced by gender-related factors such as behaviour, as well as genetics and hormones.^57,167,187^ Therefore, there is still an ongoing debate about sex-related differences in oncological outcomes for patients with metastatic RCC. Pooled meta-analyses may not fully capture the nuanced interactions of sex and ICI response. Further investigation into possible sex-based differences in the immune response to ICIs is essential for identifying patients who are most likely to benefit from particular ICI-based combination therapies.

## Summary and conclusion

Investigations of the pathobiology of sex steroid hormones and their receptors in RCC significantly expand our understanding of crucial aspects of RCC development and progression. Currently, the molecular role of SH in RCC remains to be elucidated to provide a precise model of hormonal interactions with oncogenesis. Unfortunately, the limited number of publications in this area suggests that there is a lack of research priority.

As a physiological fact, variable expression of steroid receptors has been noted when comparing normal kidney tissue and RCC tissue, which is to be seen as the potential basis for the discovered differences in the development of RCC. SH signalling in RCC suggests a not yet fully understood multifaceted dual role, influencing processes such as proliferation, invasion, apoptosis and angiogenesis through distinct molecular mechanisms. In this review, we presented comprehensive examples of both the oncogenic and tumour-suppressive effects of SH in RCC. For instance, E2 has been observed to interact with various signalling pathways, including VEGF/HIF2α, PI3K/AKT/MMP-9, TGF-β1/SMAD3 and ER-β/ANGPT-2/Tie-2, primarily through ER, whereas the activation of the AR has been linked to pathways involving PI3K/AKT → NF-κB → CXCL5, AR-c-Myc and STAT5 regulation.

Recent research has emphasized the critical role of ncRNAs in various biological functions and their profound impact on cancer, including RCC. Notably, the substantial influence of SH on the expression and functionality of numerous ncRNAs involved in the complex process of RCC development has been highlighted. Although our understanding of ncRNA function in RCC is evolving, this review underscores their potential role in initiating, promoting and progressing RCC through different miRNA/target gene axes via activation of AR and ER. Their contribution to RCC development shown in this review highlights their importance as potential therapeutic targets and biomarkers for more effective RCC therapies. However, our understanding of ncRNA function in RCC, particularly in relation to steroid hormones, remains limited, and further research is needed to explore their full functional spectrum.^188,189^

The approval of a wide range of immunotherapies is considered to be the most significant breakthrough in the treatment of advanced RCC in recent years. However, it is to be noted that many exploratory studies investigating hormone manipulation as a potential strategy for the treatment of metastatic RCC have also shown promising clinical results. This highlights the need to investigate whether ncRNAs can modulate the immune pathways involved in RCC, particularly in the context of SH involvement.

Notably, there is a lack of specific data or evidence on the interaction between SH and the efficacy of immunotherapeutic interventions in non-ccRCC subtypes due to the lower prevalence of pRCC and chRCC. As a result, the existing body of evidence does not adequately address the potential impact of SH on carcinogenesis or TME in this particular subset of RCC. Further investigation is warranted to fully elucidate this relationship in patients with non-ccRCC.

In summary, the interactions between SH and the intricate pathways within RCC are complex and not fully elucidated. Nevertheless, these findings suggest that SH plays a role in the differential response rates observed in male and female RCC patients. This review aims to rekindle interest in studying steroid hormones and their receptors in RCC, as they hold promise for therapy and biomarker development. Conducting sex-specific research, especially in the context of clinical treatments, is crucial, highlighting the importance of in-depth scientific exploration in this field.

## Declarations

**Ethics approval and consent to participate:** Not applicable.

**Consent for publication:** Not applicable.

**Availability of data and materials:** Not applicable.
